# Metabolic Dysfunction and Altered Mitochondrial Dynamics in the Utrophin-Dystrophin Deficient Mouse Model of Duchenne Muscular Dystrophy

**DOI:** 10.1371/journal.pone.0123875

**Published:** 2015-04-10

**Authors:** Meghna Pant, Danesh H. Sopariwala, Naresh C. Bal, Jeovanna Lowe, Dawn A. Delfín, Jill Rafael-Fortney, Muthu Periasamy

**Affiliations:** 1 Department of Physiology and Cell Biology The Ohio State University, Columbus, OH 43210, United States of America; 2 Department of Molecular & Cellular Biochemistry, College of Medicine, The Ohio State University, Columbus, OH 43210, United States of America; University of Minnesota, UNITED STATES

## Abstract

The utrophin-dystrophin deficient (DKO) mouse model has been widely used to understand the progression of Duchenne muscular dystrophy (DMD). However, it is unclear as to what extent muscle pathology affects metabolism. Therefore, the present study was focused on understanding energy expenditure in the whole animal and in isolated extensor digitorum longus (EDL) muscle and to determine changes in metabolic enzymes. Our results show that the 8 week-old DKO mice consume higher oxygen relative to activity levels. Interestingly the EDL muscle from DKO mouse consumes higher oxygen per unit integral force, generates less force and performs better in the presence of pyruvate thus mimicking a slow twitch muscle. We also found that the expression of hexokinase 1 and pyruvate kinase M2 was upregulated several fold suggesting increased glycolytic flux. Additionally, there is a dramatic increase in dynamin-related protein 1 (Drp 1) and mitofusin 2 protein levels suggesting increased mitochondrial fission and fusion, a feature associated with increased energy demand and altered mitochondrial dynamics. Collectively our studies point out that the dystrophic disease has caused significant changes in muscle metabolism. To meet the increased energetic demand, upregulation of metabolic enzymes and regulators of mitochondrial fusion and fission is observed in the dystrophic muscle. A better understanding of the metabolic demands and the accompanied alterations in the dystrophic muscle can help us design improved intervention therapies along with existing drug treatments for the DMD patients.

## Introduction

Duchenne muscular dystrophy (DMD) is an X-linked disease and affects 1 in 3500–6000 boys in United States of America [1–2]. The disease is caused by a mutation in the gene encoding dystrophin that is required to maintain structural integrity of striated muscle membrane [3]. As a result of the mutation, the muscle membrane is fragile and leaky, which leads to severe muscle weakness and fatigue. Symptoms appear in early childhood and death usually results by the third or fourth decade. To understand the disease pathogenesis and design therapies, the mouse model *mdx* that carries a mutation in the dystrophin gene, has been vastly used [4]. The *mdx* mice exhibit a milder phenotype of the disease as a result of the compensation by a paralogous protein called utrophin [5]. Hence, a more clinically comparable model of DMD was created by additionally knocking out the utrophin gene in *mdx* mice leading to utrophin-dystrophin deficient (DKO) mice [6].

The DKO mouse model closely mimics the human disease and may therefore be better suited to understand the disease progression and to identify therapeutic targets. Since it was first made in 1997 [6], a majority of the studies have focused on muscle structure and function [7–10]. It was shown that DKO mice exhibit severe muscle dystrophy, weakness, compromised force generation and they usually die by 20 weeks of age. The skeletal muscle is also a major site of metabolism and contributes significantly to the basal metabolic rate [11–13]. Alterations in muscle metabolism are known to cause metabolic disorders such as obesity [14] while metabolic disorders can impair skeletal muscle regeneration process [15]. Therefore; the pathogenesis of DMD is not limited to skeletal muscle but could potentially affect whole body energy expenditure, which in turn can accelerate disease progression. Despite this, the impact of DMD on muscle metabolism is not understood well. Previous studies have shown that there is an increase in the amount of oxidative fibers in DKO mice [7, 9, 16] but how it affects metabolism is unknown. Moreover, the published data is rather conflicting; studies from *mdx* mice suggest that in isolated mitochondria oxidative phosphorylation is compromised [17–21], whereas one study suggests that the overall energetics are not impaired in *mdx* mice [22]. In addition recent studies point out that there is high rate of protein turnover in *mdx* mice leading to increase in energy expenditure [23]. Hence, the above studies mostly performed on *mdx* mice so far have been inconclusive and it remains unclear how muscular dystrophy impacts muscle metabolism and whole body energy expenditure.

Therefore, the main objective of this study was to investigate metabolic alterations of the DKO mice at whole animal level and in isolated muscle. In this study we determined 1) whole body energy expenditure using indirect calorimetry, 2) oxygen consumption and fatigue resistance in isolated fast twitch extensor digitorum longus (EDL) muscle, 3) substrate utilization during muscle contraction, 4) biochemical alterations in metabolic enzymes and regulators of mitochondrial fusion-fission. Our studies suggest that muscular dystrophy increases energy demand as indicated by increased oxygen consumption relative to work done in the whole animal and in isolated muscle. Interestingly, even though the fast twitch EDL muscle generates less force, it exhibits increased fatigue resistance and shows increased potentiation of force with pyruvate. In addition, we show altered expression in key glycolytic enzymes and proteins involved in mitochondrial fission-fusion. These data collectively suggest that muscular dystrophy alters muscle energetics, mitochondrial dynamics and impacts whole body energy expenditure.

## Methods

### Ethical Approval

All study protocols were approved by the Ohio State University Institutional Animal Care and Use Committee (OSU-IACUC). All of the animal procedures were conducted in accordance with the Guide for the Care and Use of Laboratory Animals.

### Mice

*Utrn* ^*+/-*^ *;mdx (utrophin* ^*+/-*^ *;mdx)* heterozygote mice were bred to produce *utrn* ^*-/-*^ *;mdx* (utrophin-dystrophin deficient mice- DKO) as described previously [6]. Age matched wild type C57BL/10 (WT) mice were used as controls. The mice were housed at 23°C with 12:12-hour light: dark cycle and had access to food and water ad libitum. All experiments were done on 8 week-old male mice unless otherwise indicated.

### Measurement of basal metabolic rate

Comprehensive Laboratory Animal Monitoring System (CLAMS) from Columbus Instruments was used for measurement of basal metabolic parameters as published earlier [11]. Briefly, the mice were weighed and housed in individual cages at 28°C in the CLAMS set up with food and water *ad libitum*, for 48 hours. Food intake was monitored during those 48 hours by giving a measured amount of food at the beginning. The CLAMS set up is equipped to measure the % of oxygen and CO_2_ in the air entering and leaving the individual cages. Using this information the system calculates the volume of oxygen consumed (VO_2_), CO_2_ released and respiratory exchange ratio [24] (VCO_2_/ VO_2_) of the DKO mice and their WT age matched controls. The measurements were performed at 28°C, since there is minimal cold stress imposed by the environment allowing a more accurate measure of the true resting metabolic rate of the mice [25]. The physical activity (horizontal and vertical) of each mouse was monitored using a multidimensional infrared light detection system placed on bottom and top levels of each individual cage of the CLAMS. Mouse movement results in a break in the infrared beam and was counted as single activity unit.

### Western blotting

Using immunoblots we determined the levels of hexokinase 1 and 2 (HK 1 and 2), lactate dehydrogenase (LDH), pyruvate kinase M1 and M2 (PK M1and M2), Glyceraldehyde 3 phosphate dehydrogenase (GAPDH), mitochondrial electron transport chain [26] subunit proteins, mitofusin 2 (Mfn 2), dynamin related protein (Drp1), citrate synthetase (CS), mitochondrial transcription factor A (TFAM), AMP activated kinase (AMPK), phosphorylated AMPK (pAMPK) in the muscles of DKO and WT mice. Tissue homogenates were separated using standard SDS—PAGE gels; transferred to nitrocellulose membrane and immunoprobed with specific primary antibodies; HK 1, HK 2 PK M1, PK M2, AMPK, pAMPK (glycolysis antibody sampler kit -8337 (1:1000)), AMPKα, p-AMPKα (Cell signaling AMPK and ACC sampler kit -9957 (1:1000)); GAPDH (Fitzgerald 10R-G109A (1:5000)), LDH (Santa Cruz sc33781 (1:2000)), Mfn 2 (Santa Cruz sc50331 (1:500)), TFAM (Santa Cruz sc23588 (1:500)), (Mitochondrial ETC proteins- (Mitobiosciences MS604 (1:500)), CS, (Abcam ab129095 (1:1000)) Drp 1 (Abcam ab56788 (1:1000)) followed by horseradish peroxidase-conjugated secondary antibody (1:25,000–1:50,000). The antibody dilutions were as per manufacturer’s protocol. Signals were detected by WestDura substrate (Pierce) and quantified by densitometry (ImageJ 1.41o program). GAPDH was used as loading control for all the quantifications.

### Isolated muscle contractile studies

Isolated EDL from 8 week old WT and DKO mice (4 males and 3 females for each WT and DKO) was used for *ex vivo* contractile studies in a TIOX tissue bath system (Hugo Sachs Electronik-Harvard Apparatus, March Hugstetten, Germany), which allows measurement of muscle tension and dissolved oxygen simultaneously. EDL muscle was immediately harvested from mice following euthanization by CO_2_ and transferred to Tyrode buffer (121mM NaCl, 5mM KCl, 1.8mM CaCl_2_, 0.4mM NaH_2_PO_4_, 0.5mM MgCl_2_, 24mM NaHCO_3_, and 0.1mM EDTA). The buffer contained 10mM glucose as substrate and was bubbled with 95% oxygen and 5% CO_2_ while the muscle was tied at the myotendonous junction using 5/0 surgical silk. The muscle was then mounted in the TIOX tissue bath [27] containing oxygenated tyrode buffer with 10mM glucose and maintained at 30°C. The muscle was adjusted to optimal length (L_o_) for twitch production, followed by equilibration for 20 minutes. The force-frequency measurements were then made at 10, 30, 50, 70 and 100 Hz, 0.2ms pulse width, 200ms pulse train, 1 pulse train every 2 minutes. The substrate was then changed to 10mM pyruvate and allowed to equilibrate for 30 minutes followed by a second force-frequency protocol. Finally, after 30 minutes of rest a 10 minutes fatigue protocol (100Hz, 100ms pulse train, 1 pulse train every 4 seconds) was performed, followed by 8 minutes of rest (no stimulation). The specific force (Ncm^-2^) was calculated by normalizing absolute muscle force with muscle cross sectional area (CSA). CSA is given by (muscle mass)/ (L_o_ x muscle density x 0.44). 0.44 represents the ratio of muscle fiber length to optimal length for the EDL muscle [28]. Muscle density is 1.06 gcm^-3^. The oxygen consumption was recorded during the fatigue protocol and baseline oxygen without muscle was also recorded at the end of the experiment. The difference between the two was used to determine the rate of oxygen consumption by the muscle [27]. The calculations for oxygen consumption measurements are as follows:

The solubility constant of oxygen in water at 30°C is 0.001203 molL^-1^atm^-1^ [27]. Converting units of solubility constant from molL^-1^atm^-1^ of oxygen to μlml^-1^mm Hg^-1^ ((0.001203 X 32 X 1000)/(760 X 1.428) where, 32 is mol wt of O_2_, 760 is mm Hg for 1 atm and 1.428 is density of oxygen) gives- 0.035471031 μlml^-1^mm Hg^-1^.

Now total oxygen consumed by muscle in 600 seconds (μlsg^-1^) is given by:

Solubility constant (μlml^-1^mm Hg^-1^) X volume of bath (12.7 ml) X change in partial pressure of oxygen (mm Hg) over 600 seconds/ weight of muscle (g).

Oxygen consumed/integral force (μlg^-1^Ncm^-2^) is given by:

Total oxygen consumed in 600 seconds (μlsg^-1^)/ Force time integral (Nscm^-2^).

For a set of experiments the order of using glucose and pyruvate as the substrate was reversed. Since, the same muscle was used for force frequency measurements with glucose and then pyruvate followed by a fatigue protocol; we did not want to damage the DKO muscle as they are weak and not used to producing maximal force. Therefore, the maximum frequency during force frequency measurements was not exceeded beyond 100Hz.

### Transmission electron microscopy (TEM)

Skeletal muscle ultrastructure was analyzed using TEM. EDL muscles from 8-week old WT (3 males) and DKO (2 females 1 male) mice were excised, cut into thin slices (2mm X 1mm), and fixed in Trumps fixative (30% formaldehyde, 10% glutaraldehyde), followed by staining with uranyl acetate. Tissues were embedded in epoxy resin and sectioned for light microscopy to confirm orientation longitudinally along muscle fibers. Microtome thin sections stained with lead citrate were examined by a transmission electron microscope (JEOL ExII, Peabody, MA) at different magnifications. Representative images are shown after analyzing 8 images from each mouse.

### Statistics

Data are presented as mean ± standard error of mean. Statistical analysis was performed using the Prism 3.0 software. Student’s unpaired *t* test or 2way ANOVA was used to determine statistically significant differences. P<0.05 was considered significant.

## Results

### Oxygen consumption per unit activity is significantly higher in the DKO mice

**Fig 1:**
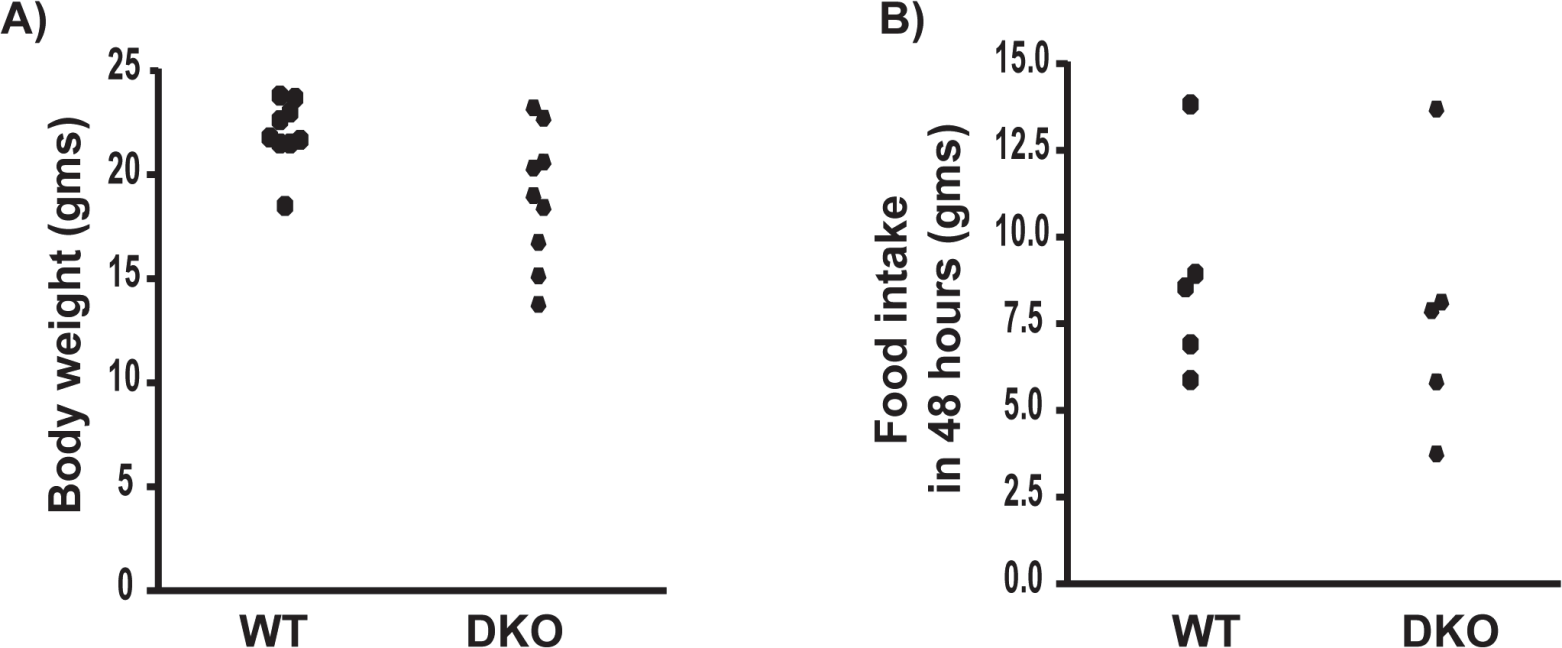
DKO mice weigh less but consume similar amount of food when compared to WT controls. A) DKO mice weigh significantly less than WT (p = 0.0298). B) Food consumption between WT and DKO mice is not significantly different.

**Fig 2:**
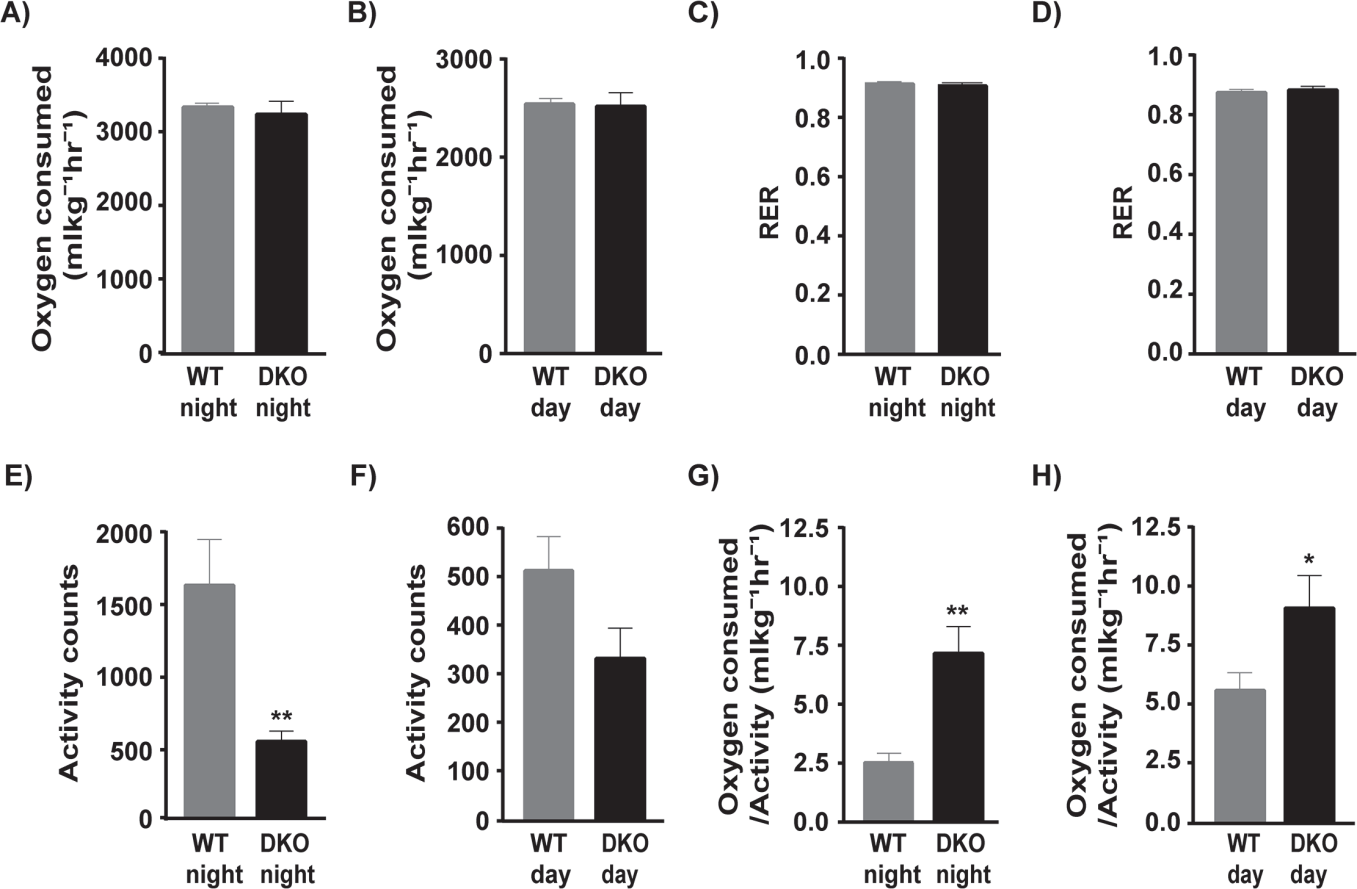
Whole body energy expenditure of WT and DKO mice. A) Rate of oxygen consumption in DKO mice is not significantly different from WT at both night and B) day. Respiratory exchange ratio (F) is similar in WT and DKO mice both at C) night and D) day. Activity counts measured in WT and DKO mice during E) night and F) day. Activity counts are significantly reduced (p = 0.0060) in DKO mice at night compared to WT. Oxygen consumption per unit activity is significantly higher in the DKO mice both during G) night (p = 0.0023) and H) day (p = 0.0463) p<0.05 = significant. * = p<0.05, ** = p<.01.

Depending on the severity of disease, there is lot of variation in the weight of DKO mice. However, they tend to weigh significantly less than the WT controls (Fig 1A p = 0.0298 WT: 21.74 ± 0.55 gm DKO: 18.96 ± 1.08 gm, n = 10 WT and n = 9 DKO). Interestingly, the food intake during 48 hours was not different between WT and DKO mice (Fig 1B). To understand the impact of muscle disease on whole body energy expenditure, we measured oxygen consumption using the CLAMS setup and determined basal metabolic rate in 8-week old DKO mice that show significant muscle pathophysiology [6–7]. Our results show that the whole body oxygen consumption in the DKO mice is not significantly different from WT controls at night (Fig 2A WT: 3336 ± 52 ml kg^-1^hr^-1^ DKO: 3239 ± 177 ml kg^-1^hr^-1^, n = 7 WT and n = 7 DKO) or day (Fig 2B WT: 2542 ± 56 ml kg^-1^hr^-1^, DKO: 2519 ± 137 ml kg^-1^hr^-1^, n = 7 WT and n = 7 DKO). In addition, the RER, which indicates the type of fuel utilized [29], is also not altered in the DKO mice during night (Fig 2C WT: 0.9128 ± 0.0082, DKO: 0.9066 ± 0.0106, n = 7 WT and n = 7 DKO) or day (Fig 2D WT: 0.8740 ± 0.0101, DKO: 0.8827 ±. 01193, n = 7 WT and n = 7 DKO).

Since mice are nocturnal, the daytime activity levels although lower in DKO mice are not significantly different between WT and DKO mice (Fig 2F WT: 512.4 ± 70.4, DKO 331.5 ± 62.5, n = 7 WT and n = 7 DKO). Interestingly, the night activity levels of the DKO mice are significantly lower (p = 0.006) than WT mice (Fig 2E WT: 1603 ± 314.5, DKO 528.6 ± 71 n = 7 WT and n = 7 DKO). As a result, the whole body oxygen consumption per unit activity is significantly higher in the dystrophic mice during the night (p = 0.0023) (Fig 2G WT: 2.522 ± 0.39 ml kg^-1^hr^-1^, DKO: 7.161 ± 1.133 ml kg^-1^hr^-1^, n = 7 WT and n = 7 DKO) and during day (p = 0.0463) (Fig 2H WT: 5.578 ± 0.746 ml kg^-1^hr^-1^, DKO: 9.066 ± 1.381 ml kg^-1^hr^-1^, n = 7 WT and n = 7 DKO).

### Isolated EDL muscle from DKO mice consumes more oxygen per unit integral force

**Fig 3:**
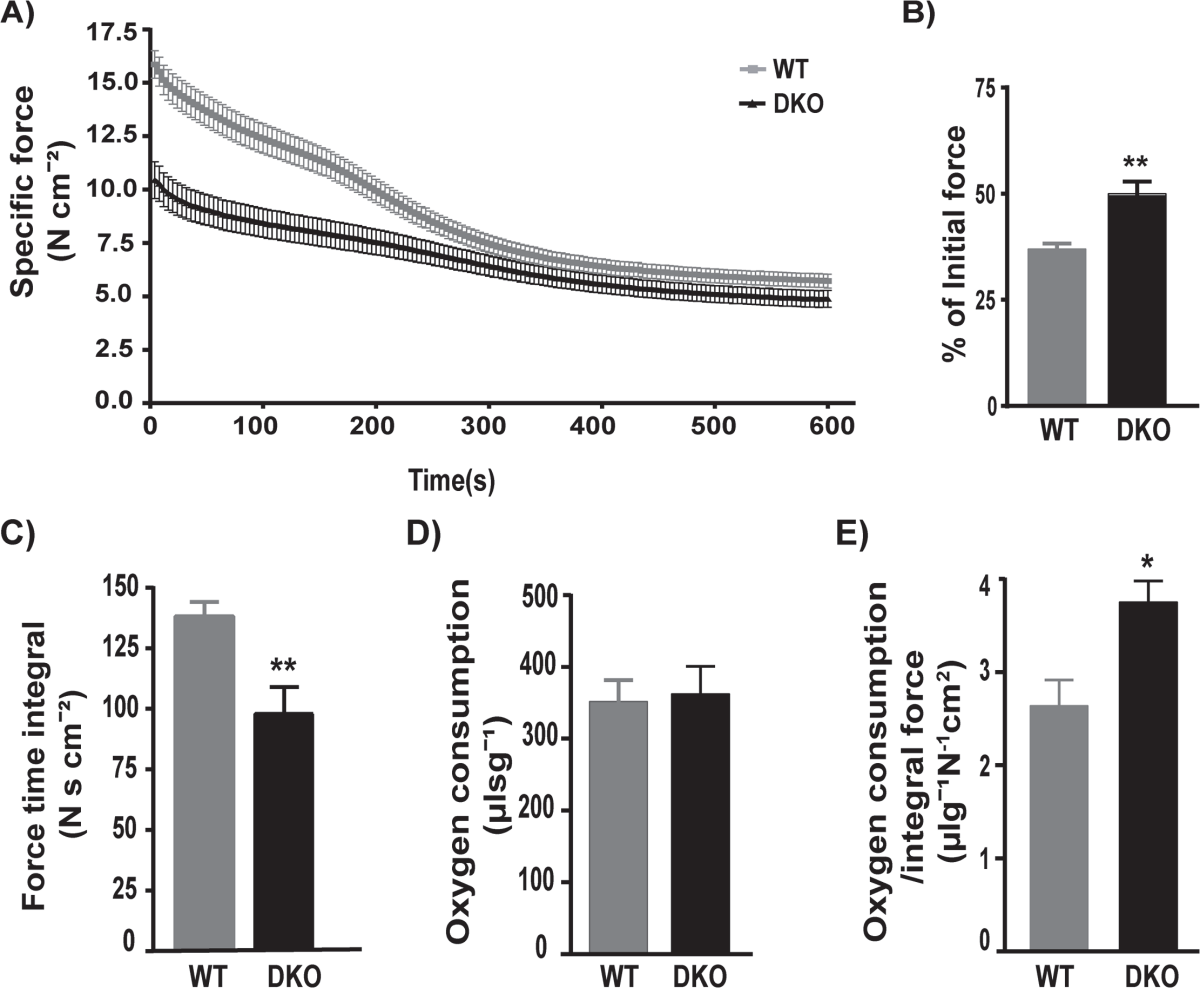
Increased oxygen consumption relative to integral force. A) The fatigue profile of WT and DKO EDL during the 10 minutes fatigue shows that DKO EDL generates lesser force and fatigues less. B) The % of initial force after the 10 minute fatigue is higher in DKO EDL indicating less fatigue (p = 0.0019). C) The quantified force time integral over the entire 10 minutes fatigue protocol is significantly reduced in the DKO EDL compared to WT (p = 0.0097). D) Oxygen consumption over 10 minutes fatigue is not significantly different in WT and DKO EDL muscle. E). Oxygen consumed per unit integral force produced is significantly higher in DKO EDL compared to WT (p = 0.0110). p< 0.05 is significant. * = p<0.05, ** = p<0.01.

Previous studies have shown that soleus a slow twitch muscle from DKO mice generates less force but is more fatigue resistant [7]. However DMD affects the fast twitch glycolytic muscles more severely than the slow twitch [30–31], therefore we investigated the contractile properties and oxygen consumption in a fast twitch muscle, EDL, using the TIOX bath system. We chose EDL as a representative of fast twitch muscles because it is small enough to measure contractile properties in an isolated system. Our data shows that the EDL muscle from DKO mice produces less force and fatigues less over the 10-minute protocol (Fig 3A and 3B p = 0.0019, WT: 36.98± 1.29, DKO: 50.05± 2.84, n = 6 WT and n = 6 DKO). The DKO EDL exhibits a significantly lower force time integral than WT over the 10-minute fatigue period (p = 0.0097) (Fig 3C. WT: 138.1± 6.01 N s cm^-2^, DKO: 97.88 ± 11.10 N s cm^-2^ n = 6 WT and n = 6 DKO). We chose a milder fatigue protocol because we wanted to measure oxygen consumption over the 10-minute period without damaging the dystrophic muscle. Interestingly, total oxygen consumed by DKO EDL over the 10 minute fatigue protocol is similar to WT EDL (Fig 3D WT: 352 ± 29.66 μl s g^-1^, DKO 362 ± 38.42 μl s g^-1^, n = 6 WT and n = 6 DKO) resulting in significantly higher oxygen consumed per unit integral force (p = 0.0110) (Fig 3E WT: 2.639 ± 0.276 μl g^-1^N^-1^cm^2^, DKO: 3.753 ± 0.228 μl g^-1^N^-1^cm^2^, n = 6 WT and n = 6 DKO) in DKO EDL.

### The isolated EDL muscle from DKO mice produces higher force with pyruvate compared to glucose

**Fig 4:**
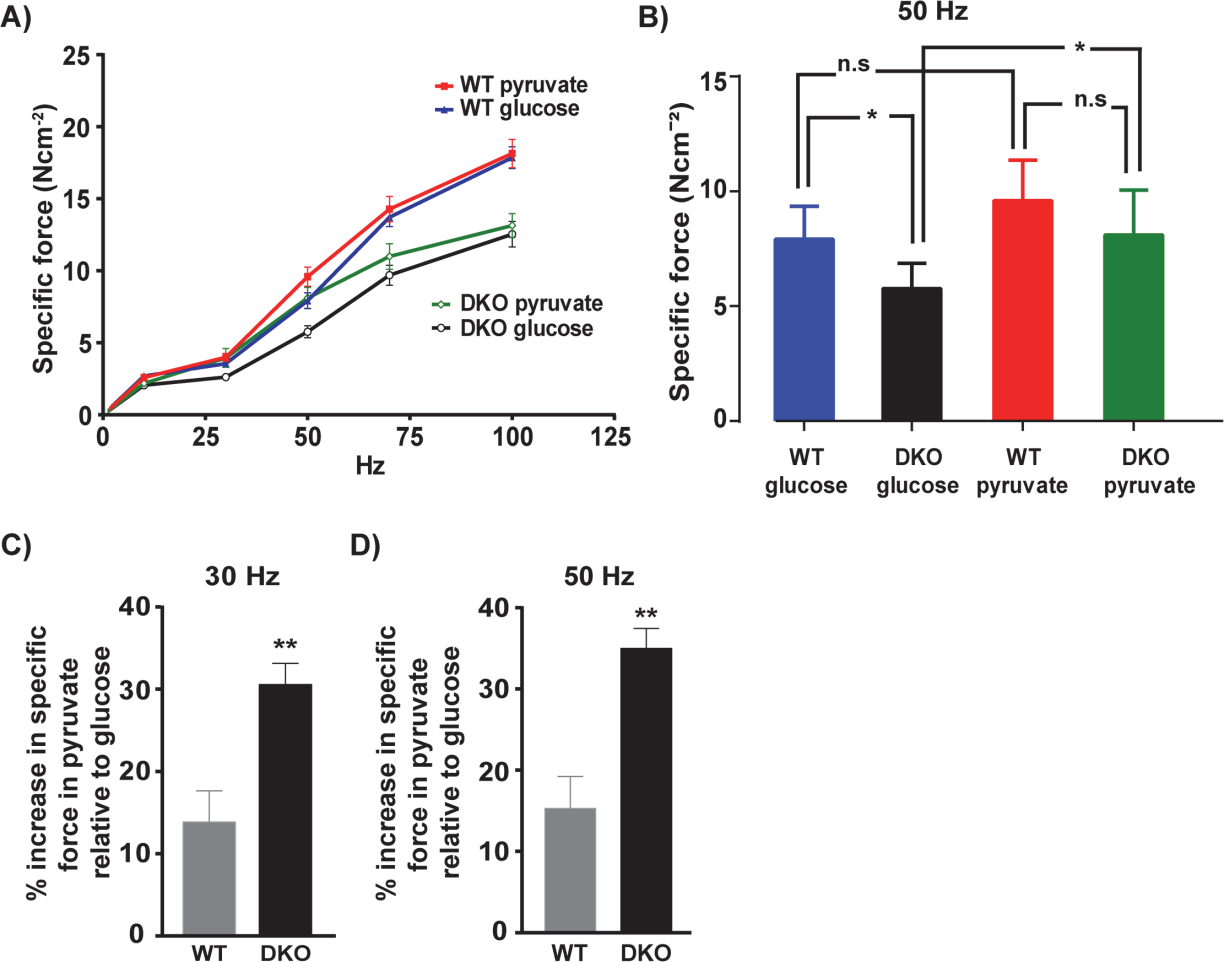
Increased potentiation of force by pyruvate in DKO EDL. A). Effect of substrate on force production showing an increase in force production using pyruvate as a substrate at lower frequencies in DKO mice. B) B) Specific force produced by WT EDL is significantly higher (p<0.05) than DKO EDL when glucose is used as a substrate at 50 Hz. In the presence of pyruvate the specific force produced by WT EDL is not significantly different from DKO EDL at 50Hz. There is a significant increase in force production in DKO EDL when pyruvate is used as a substrate compared to glucose (p<0.05). However, the force produced by WT EDL in the presence of pyruvate is not significantly different from the force produced in presence of glucose. C) % Increase in force using pyruvate as a substrate relative to glucose is significantly higher in DKO EDL compared to WT at 30Hz (p = 0.0033) and D) 50Hz (p = 0.0011). p< 0.05 is significant. * = p<0.05, ** = p<0.01.

It is known that the fast twitch glycolytic muscles like EDL prefer glucose as a substrate whereas slow twitch oxidative muscles like soleus show a greater increase in force production in the presence of pyruvate as compared to glucose [32]. Since the fast twitch muscles of DKO mice have more oxidative fibers and consume more oxygen relative to integral force produced we wanted to investigate if their substrate utilization is altered. We observed that with 10 mM glucose, the specific force produced by dystrophic EDL is significantly less than the WT control at both low and high stimulation frequency (Fig 4A and specific force shown for 50 Hz Fig 4B, p< 0.05 for WT glucose and DKO glucose (WT glucose: 7.919 Ncm^-2^, DKO glucose: 5.767 Ncm^-2^, n = 7 WT and n = 7 DKO), which is in agreement with previous studies [7]. However, when pyruvate is used as a substrate the DKO muscle produces higher force than it produces with glucose (p<0.05 Fig 4B) especially at lower frequencies and as a result the specific force produced by WT and DKO EDL is similar at 50Hz. (Fig 4B WT pyruvate: 9.593 ± 0.667 Ncm^-2^, DKO pyruvate: 8.109 ± 0.734 Ncm^-2^, n = 7 WT and n = 7 DKO). Hence, the potentiation of force by pyruvate relative to glucose is significantly higher in DKO EDL as compared to WT EDL at 30 Hz (p = 0.0033) (Fig 4C WT: 13.86 ± 3.79%, DKO: 30.57 ± 2.57%, n = 7 WT and n = 7 DKO) and 50 Hz (p = 0.0011) (Fig 4D WT: 15.31 ± 3.93%, DKO: 34.98 ± 2.45%, n = 7 WT and n = 7 DKO).

### The dystrophic muscles show an increase in hexokinase 1 and pyruvate kinase M2

**Fig 5:**
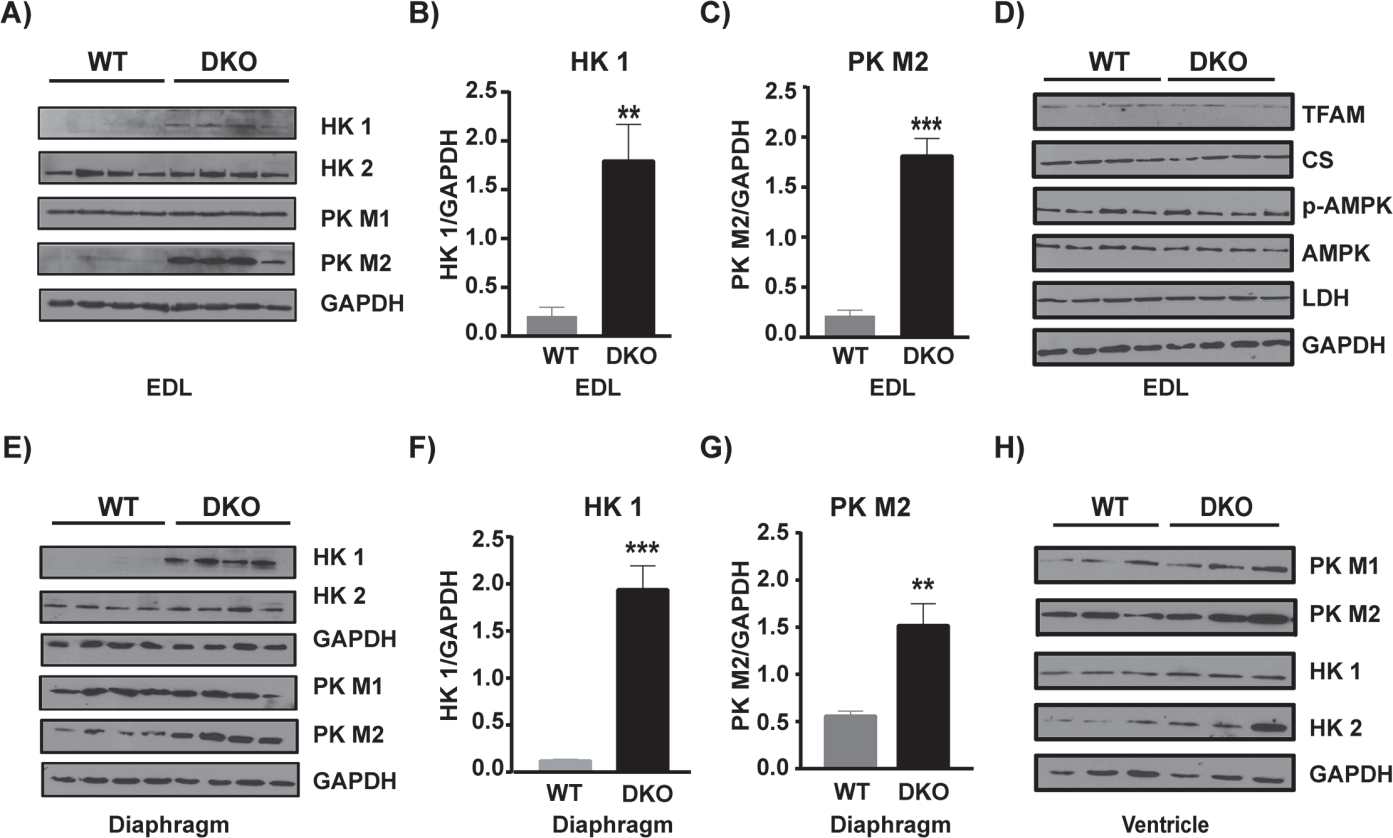
Increased expression of glycolytic enzymes in DKO muscles. A) Western blots depicting higher levels of glycolytic enzymes DKO EDL compared to WT. B) HK1 protein level normalized to GAPDH is significantly higher in DKO EDL compared to WT (p = 0.0063) C) PK M2 protein level normalized to GAPDH is significantly higher in DKO EDL compared to WT (p = 0.0001). D) Western blot showing key metabolic regulators are unchanged in DKO EDL compared to WT. E) Western blots showing glycolytic enzymes protein levels are higher in DKO diaphragm. F) HK1 protein level normalized to GAPDH is significantly higher in DKO diaphragm compared to WT (p = 0.0004) and G) PK M2 protein level normalized to GAPDH is significantly higher in DKO diaphragm compared to WT (p = 0.0066). H). Glycolytic enzymes protein expression levels in ventricle. p< 0.05 is significant. * = p<0.05, ** = p<0.01 and *** = p<0.001. HK 1- Hexokinase 1, HK 2- Hexokinase 2, PK M1- Pyruvate kinase M1, PK M2- Pyruvate kinase M2, CS- Citrate synthetase, TFAM- Mitochondrial transcript factor A, LDH- Lactate dehydrogenase.

Next we wanted to determine if disease progression has significantly altered metabolic pathways utilizing glucose or fatty acids as well as mitochondrial metabolism. Since we measured the contractile properties of the EDL muscle, we first analyzed this muscle for metabolic protein levels. Our western blotting analyses of EDL show a switch in hexokinase (HK) isoform expression. HK regulates the first step in glucose metabolism and transfers the phosphate group from ATP to glucose. HK has 2 isoforms- HK 1 is localized to mitochondria while HK 2 is found both in cytosol and associated with mitochondria. The glucose phosphorylated by HK 1 is usually driven towards glycolysis, while that by HK 2 is directed towards glycogen synthesis [33]. We observe an 8.7 fold increase in HK 1 in the dystrophic EDL muscle (Fig 5A and 5B p = 0.0063, n = 4 WT and n = 4 DKO). Additionally we found that the glycolytic enzyme pyruvate kinase M2 (PK M2) is significantly upregulated (8.4-fold) in dystrophic muscle (Fig 5A and 5C, p = 0.0001, n = 4 WT and n = 4 DKO). PK catalyzes the last step of glycolysis and transfers the phosphate from phosphoenolpyruvate (PEP) to ADP thereby releasing ATP and pyruvate. It has 2 isoforms, PK M1 is usually expressed in skeletal muscle and has high affinity for its substrate, and PK M2 that is not detected in adult muscle fibers has a lower affinity for its substrate and has recently been shown to be a marker of proliferating cells [34]. Other key metabolic markers analyzed including AMP-Kinase, (both AMPK and p-AMPK), citrate synthetase and lactate dehydrogenase (LDH) did not show any significant difference (Fig 5D). Since diaphragm and ventricle are muscle groups severely affected in DKO mice, we also studied the HK and PK protein levels in them. Diaphragm showed 16-fold increase in HK 1 (Fig 5E and 5F, p = 0.0004, n = 4 WT and n = 4 DKO) and 2.7-fold increase in PK M2 (Fig 5E and 5G, p = 0.0066, n = 4 WT and n = 4 DKO), while ventricle did not show a significant increase in HK1 or PK M2 (Fig 5H).

### Increased expression of Mfn 2 and Drp 1 indicates increased fusion/fission of mitochondria in dystrophic muscle

**Fig 6:**
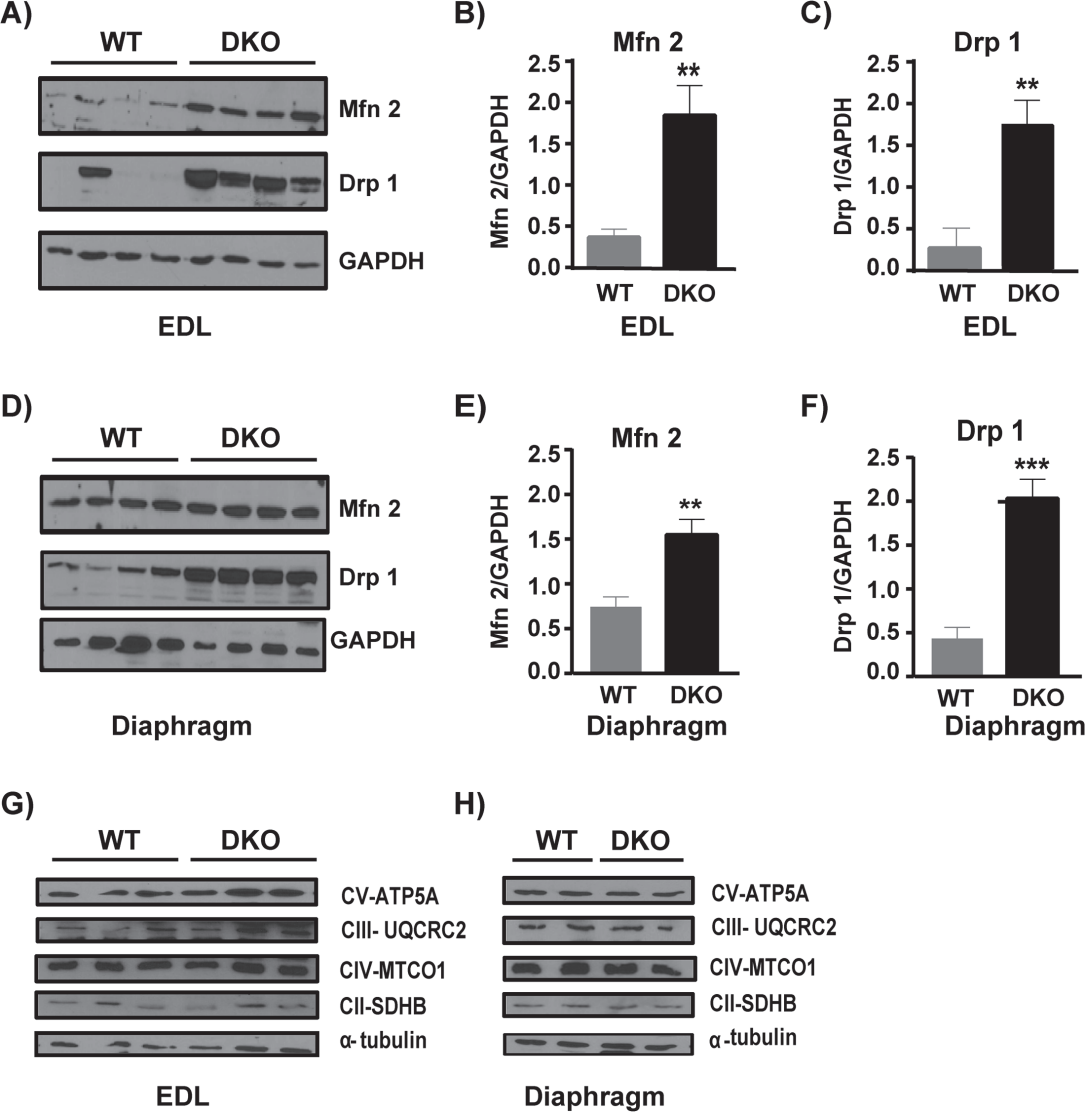
Increased expression of mitochondrial fusion and fission regulators in DKO muscles. A) Western blots depicting mitochondria fission (Drp 1) and fusion (Mfn 2) regulators in WT and DKO EDL. B) Mfn 2 protein level normalized to GAPDH is significantly higher in DKO EDL compared to WT (p = 0.0073). C) Drp 1 protein level normalized to GAPDH is significantly higher in DKO EDL compared to WT (p = 0.0089). D) Western blots depicting mitochondria fission (Drp 1) and fusion (Mfn 2) regulators in WT and DKO diaphragm. E) Mfn 2 protein level normalized to GAPDH is significantly higher in DKO diaphragm compared to WT (p = 0.0074). F) Drp 1 protein level normalized to GAPDH is significantly higher in DKO diaphragm compared to WT (p = 0.0007). Western blots depicting similar mitochondrial electron transport chain complex protein levels in G) EDL and H) diaphragm of WT and DKO mice. p< 0.05 is significant. * = p<0.05, ** = p<0.01. Mfn 2- Mitofusin 2, Drp 1—Dynamin related protein 1, CV ATP5A- Complex V F1-F0 ATP synthase subunit, CIII UQCRC2- Complex III ubiquinol-cytochrome c reductase subunit, CIV MTCO1- Complex IV Cytochrome C Oxidase core subunit, CII SDHB- complex II succinate dehydrogenase subunit.

**Fig 7:**
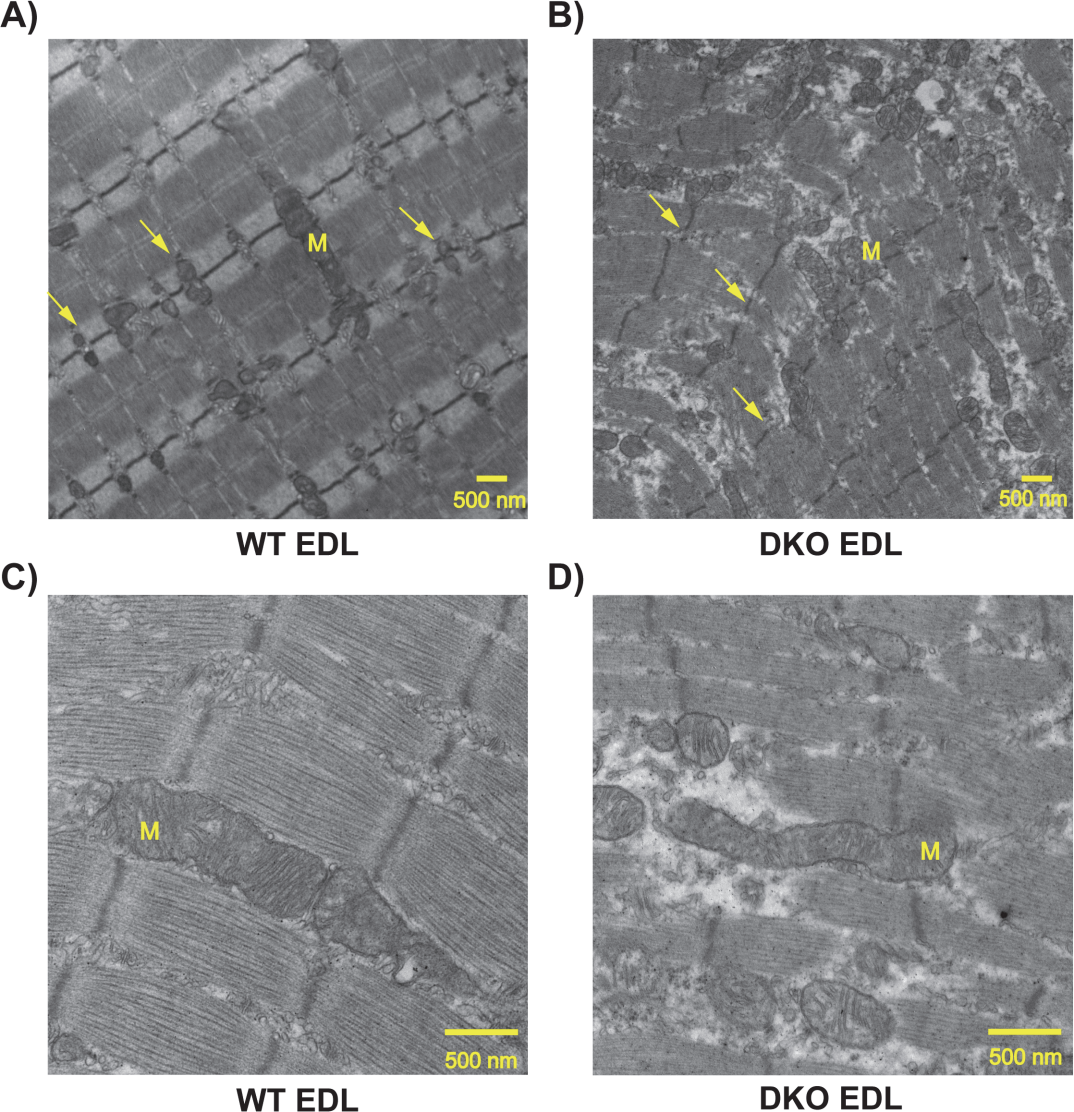
Transmission electron microscopic images show mitochondrial localization is altered in DKO EDL muscle. A) WT EDL B) DKO EDL at 14000X magnification. C) WT and D) DKO at 34000x magnification. The arrows point to the localization of mitochondria (M) which are at the I band on either side of the Z disc in WT, but this tight localization is reduced in the DKO EDL.

Since energy expenditure and substrate utilization is altered in the DKO mice we wanted to determine whether there was a change in regulators of mitochondrial fusion-fission and/ or mitochondrial localization. The protein levels of Mfn 2, a regulator of mitochondrial fusion [35–36], are 5-fold higher (Fig 6A and 6B, p = 0.0073, n = 4 WT and n = 4 DKO) and the mitochondrial fission regulator Drp 1 [37] are 6-fold higher in the dystrophic EDL (Fig 6A and 6C p = 0.0089, n = 4 WT and n = 4 DKO) suggesting increased fusion and fission events in diseased muscle. In the DKO diaphragm Mfn 2 is 2-fold higher (Fig 6D and 6E p = 0.0074, n = 4 WT and n = 4 DKO) and Drp 1 is 4.6-fold higher (Fig 6D and 6F p = 0.0007, n = 4 WT and n = 4 DKO). We next analyzed the expression level of electron transport chain [26] enzymes and found that the expression of ETC proteins is unaltered in both EDL and diaphragm of DKO mice (Fig 6G and 6H). Similarly the expression of TFAM, a transcription factor involved in mitochondrial DNA replication and repair is unaltered (Fig 5D). Additionally we analyzed mitochondrial localization and distribution in EDL muscles by TEM. The normal arrangement of mitochondria at the I band on either side of the Z disc seen in WT EDL (Fig 7A and 7C) is not observed in DKO EDL (Fig 7B and 7D).

## Discussion

The DKO mouse model has been widely used as an animal model to understand the progression of DMD and designing strategies to rescue muscle function [26, 38–39]. Although many studies have been carried out on the DKO mice, there is a paucity of data on how the disease process affects energy expenditure at the whole animal and at the muscle level. In this study we investigated the metabolic characteristics at the whole animal and in isolated fast twitch muscle of the DKO mice. We focused our studies on EDL because DMD affects fast twitch muscles more severely [30–31] and EDL muscle is better suited to simultaneously measure contractile properties and oxygen consumption using a TIOX bath. The data presented here demonstrate that muscle energetics and mitochondrial dynamics are severely affected and multiple metabolic adaptations are induced to maintain energy homeostasis in dystrophic muscle. Although, DKO mouse model is a better phenotypic mouse model for DMD, the findings from our study need to be confirmed in human patients and muscle biopsies, which is beyond the scope of this project.

### Dystrophic muscle relies on higher oxygen consumption to meet its energy demand

Previous metabolic studies on DMD were focused mostly on *mdx* mice and remain inconclusive [17, 20, 22–23]. Even though *mdx* mice carry the same genetic defect as DMD patients, they show a much milder phenotype and have an almost normal life span [40]. The DKO mice on the other hand, show a clinically similar phenotype to DMD patients and are better suited to study the metabolic regulation in muscle dystrophy. A hallmark of these dystrophic muscles is the switch from fast to slow fiber types and a concomitant shift to a more oxidative metabolism [9]. In the present study we wanted to understand the impact of dystrophy on whole body energy expenditure using the CLAMS set up. A major finding of this study is that the DKO mice consume significantly higher oxygen, when normalized to physical activity levels. In addition, even though DKO mice are smaller in size and weigh less they consume similar amount of food. However, it should be noted that the mice tend to spill the food in the CLAMS cages and so the food intake measurements have a lot of variation. Interestingly we found that the isolated DKO EDL muscle also consumed more oxygen relative to force produced. A potential explanation for this higher oxygen consumption relative to activity or force could be due to either an inefficiency in energy utilization or a decrease in energy production by mitochondria as has been shown previously in *mdx* mice [41].

Another key finding is that the DKO EDL fatigues less, which is probably because it generates less force over the 10-minute fatigue protocol as compared to WT (Fig 2B and Fig 3A), [7]. However, fatigue resistance has been previously observed for DKO soleus [7], which was suggested to be a result of increased oxidative fibers and switch in myosin isoforms. The number of slow oxidative fibers is also increased in DKO EDL [7] and that could be responsible for the increase in fatigue resistance EDL. Previous studies have shown that there is switch in the myosin isoform expression with an increase in myosin heavy chain 1 (MHC 1) and MHC 2a fibers and a concomitant decrease in MHC 2b fibers in fast twitch glycolytic muscles including the EDL of the DKO mice [7, 42]. Schneider et al., further documented an increase in the slow sarco(endo)plasmic reticulum calcium ATPase (SERCA) 2a isoform and a reduction in the fast SERCA 1a in the fast twitch glycolytic muscles. This was associated with an upregulation of Sarcolipin (SLN), which is a regulator of SERCA [43–44] and a decrease in SERCA mediated calcium uptake [42]. It is well known that apart from regulating force generation, increase in cytosolic calcium can directly upregulate mitochondrial oxidative metabolism and other metabolic pathways regulated by calcium mediated signaling [45–46]. Hence, alterations in calcium cycling and an increase in slow oxidative fibers could potentially convert the fast twitch EDL to a slow oxidative soleus like muscle. The activation of a slow oxidative myofiber program in a fast glcyolytic muscle has been suggested previously to be beneficial in coping with DMD [47–48].

### Dystrophic EDL, a fast twitch glycolytic muscle performs better with pyruvate

In comparison to glucose as a substrate, the DKO EDL exhibits greater potentiation of force when pyruvate is used as a substrate. This preference in substrate utilization is intriguing since CLAMS data indicate that the DKO and WT mice have similar RER suggesting no alteration in terms of whole body fuel utilization. Previously this potentiation in response to pyruvate has been observed in soleus but not in a fast twitch glycolytic muscle like EDL [32]. It has been shown that in slow oxidative soleus, which has high resting inorganic phosphate (Pi) levels, pyruvate increases ATP production and decreases Pi, which in turn can increase myosin ATPase activity and force [49]. However, in fast twitch glycolytic muscles, resting Pi levels are low, so using pyruvate as a substrate does not potentiate force. However an increase in the number of oxidative fibers in DKO muscle could lead to higher resting Pi levels [9, 22] and could potentially explain the increase in potentiation of force by pyruvate. Alternatively, the positive ionotropic affect of pyruvate on cardiac muscle has been demonstrated to be a result of increased calcium sensitivity of myofilaments [50]. It has been shown that in DKO skeletal muscles there is an upregulation of the cardiac/ slow troponin isoform along with induction of other slow fiber type genes, like SERCA 2a, SLN and MHC1 [7, 16, 42]. Hence, increase in calcium sensitivity of myofilament could be another mechanism of increased force production in pyruvate at low frequencies in the DKO EDL but not in the WT EDL.

### Dystrophic muscles show altered mitochondrial dynamics as evidenced from increased expression of Mfn2 and Drp1 and distribution of mitochondria

An exciting finding of this study is that proteins involved in fusion and fission (Mfn 2, Drp 1) of mitochondria were upregulated several fold in DKO muscles. Expression of mitochondrial fusion regulator, Mfn 2, is often associated with states of increased energy expenditure like exercise and cold induced thermogenesis [35, 51] and it helps in coping with the higher metabolic demand by increasing mitochondrial based energy production [52]. Our data shows that there is increase in oxygen consumption relative to work done in DKO mice as well as in isolated EDL muscle suggesting a higher metabolic demand in DKO mice. It has also been shown in *mdx* mice that there is an inefficiency in energy production by mitochondria [41]. Hence upregulation of Mfn 2 in DKO muscle could be an adaptation to increase energy production in a diseased muscle to meet the higher metabolic demand. However, it is also possible that the increase in Mfn 2 expression is a result of regenerating muscle fibers [53]. High levels of Drp 1 in the DKO muscles suggest that there is increased mitochondrial fission, which could be a consequence of uncoupled mitochondrial activity [54–55]. An increase in Drp 1 has been shown to be important for removal of impaired/uncoupled mitochondria that can otherwise produce more reactive oxygen species [56] and cause further damage [57]. The dystrophic muscles also exhibit elevated oxidative stress and increased muscle damage [6, 9, 58–59] and upregulation of Drp1 could be beneficial for removing unhealthy mitochondria. Therefore, the increase in mitochondrial fusion-fission regulators could potentially be an adaptive response of the DKO muscle to the increased metabolic stress. In addition our electron microscopy data indicates that the tight localization of mitochondria at the Z line is reduced in the DKO EDL, which could also lead to inefficiency in energy supply to the myofilaments. Collectively our data along with previous study done on *mdx* mice suggest that the higher metabolic needs of DKO mice as indicated by increased oxygen consumption relative to work done, is most likely due to a combination of inefficiency in energy production and altered distribution of mitochondria [41].

### Upregulation in key glycolytic regulators suggest altered metabolic demand in DKO muscles

The inefficiency in oxidative phosphorylation along with the constant damage and repair that is seen in DKO muscle places a tremendous metabolic stress on these muscles. In such a situation the diseased muscle would have to rely on increased glycolysis to meet its energy demands. In this regard earlier studies in DMD patients show alterations in HK 2 levels [60–61]; an increase in pyruvate kinase activity was also reported although changes in isoform expression were not analyzed [62]. Our studies for the first time show a significant upregulation in key glycolytic enzymes, especially HK 1 and PK M2. HK 1 activity increases glycolysis and PK M2 reduces pyruvate build up thus preventing back inhibition of glycolysis [34]. This suggests the possibility of increased glycolytic activity to cope up with the energetic needs of the diseased muscle. Alternatively, the combined effect of these two enzymes could also lead to increased glycolytic intermediates, which can be used as building blocks for protein synthesis. Since dystrophic muscles have high rates of protein turnover [23], these glycolytic intermediates could play an important role in replenishing the amino acid pool. Another possible explanation for increased glycolytic enzyme expression in the DKO muscle could be the presence of more regenerating fibers since proliferating cells rely more on glycolysis [63]. Future studies are needed to measure the metabolite levels and glycolytic enzyme activities to confirm that glycolytic flux is enhanced in the DKO muscles.

In summary this study shows for the first time the impact of muscular dystrophy on whole body energy expenditure and its effect on muscle performance, especially on fast twitch glycolytic muscle. Collectively our data and published studies [7, 9, 42] show that dystrophic muscle (especially fast twitch), which requires high energy supply due to muscle damage and oxidative inefficiency, tries to compensate by 1) fiber switching to reduce force and muscle damage, 2) increasing mitochondrial fission-fusion to boost energy production and 3) increasing glucose metabolism to compensate for energy needs and provide building blocks for muscle repair. In spite of this the diseased muscle is severely compromised both functionally and metabolically. Moreover, it has been recently shown that metabolic remodeling agents have beneficial effect in improving the disease phenotype in mdx mice [64]. Hence, it is imperative that we advance our understanding of the metabolic needs of dystrophic muscle so as to provide the right dietary interventions to DMD patients, which can further improve the therapeutic effects of the currently used drugs.
